# A real-time road detection method based on reorganized lidar data

**DOI:** 10.1371/journal.pone.0215159

**Published:** 2019-04-16

**Authors:** Fenglei Xu, Longtao Chen, Jing Lou, Mingwu Ren

**Affiliations:** School of Computer Science and Engineering, Nanjing University of Science and Technology, Nanjing, China; University of British Columbia, CANADA

## Abstract

Road Detection is a basic task in automated driving field, in which 3D lidar data is commonly used recently. In this paper, we propose to rearrange 3D lidar data into a new organized form to construct direct spatial relationship among point cloud, and put forward new features for real-time road detection tasks. Our model works based on two prerequisites: (1) Road regions are always flatter than non-road regions. (2) Light travels in straight lines in a uniform medium. Based on prerequisite 1, we put forward difference-between-lines feature, while *ScanID* density and obstacle radial map are generated based on prerequisite 2. According to our method, we construct an array of structures to store and reorganize 3D input firstly. Then, two novel features, difference-between-lines and *ScanID* density, are extracted, based on which we construct a consistency map and an obstacle map in Bird Eye View (BEV). Finally, the road region is extracted by fusing these two maps and refinement is used to polish up our outcome. We have carried out experiments on the public KITTI-Road benchmark, achieving one of the best performances among the lidar-based road detection methods. To further prove the efficiency of our method on unstructured road, the visual outcomes in rural areas are also proposed.

## Introduction

Traversable road detection is always a core task in the context of autonomous driving vehicles, which has been studied for decades. Bunches of implementations are proposed based on various sensors, in which vision-based road detection is the most conventional kind. However, the lack of depth information makes environmental perception inadequate to construct a perfect road model. So, a consensus has been reached that range data is necessary in road detection, which leads to researches on utilization of 3D information recently. Range data is usually provided by stereo-cameras, radars or lidars. In off-road environment, which is much more challenging compared to well-structured urban scenes, 3D lidar scanners are more widely needed.

3D point cloud processing based on lidar boom recently, due to the rapid development of lidar. The resolution of lidar starts to increase from centimeter level to millimeter level, while solid-state lidar is also being on mass production. 3D lidar scanners can capture geometry in a very high resolution, while not suffering from external illumination, which is a big trouble to image-based road detection. Although the high resolution of 3D lidar data makes it possible to detect various obstacles and traversable areas for unmanned vehicles, it brings mass calculation to searching or indexing tasks. Therefore, it is meaningful to design a model to transform the discrete and unorganized lidar point cloud into an organized form and cut computation reasonably. So, we involve a more organized lidar form with abundant information in this paper.

Traditional lidar-based road detection is constructed in view of flat road plane assumption. However, taking unstructured road into account, we believe that this assumption is fragmentary. According to our view, road region is just relatively flatter than non-road regions. Gentle ups and downs are frequently existed on road, while mild obstacles are also permitted especially in rural road scenes. This is the base, on which we put forward difference-between-lines feature and use adaptive threshold to locate road boundary point. Another prerequisite concerned is that light travels in straight lines in a uniform medium. So laser would be blocked by obstacles, which causes a high point density before a positive obstacle with an undetected region generated after it. Obstacles and the undetected regions behind pose a threat to safe autonomous driving, so we would like to describe both of them as obstacles in our work.

In this paper, we put forward a new organization mode of lidar data, which generates spatial relationship among points. *ScanID* and *PointID* are defined to directly describe this relationship, which are quantification of scanner pitch angle and lidar rotation angle to an extent. Next, two new features, difference-between-lines and *ScanID* density, are proposed based on this organized lidar data presentation, which are robust to structured as well as unstructured road detection. Finally, novel obstacle and road detection algorithms are generated by utilizing these two features respectively. Our method enjoys low cost of computing, meeting real-time requirement in the field of autonomous driving.

The rest of this paper is organized as follows. The normal existing methods and our approach are introduced in Section Materials and Methods. Section Experiments shows the experiments and corresponding results of our approach, as well as the comparison with other existing methods. We draw our conclusion in Section Conclusion. Note that this paper is best viewed in color.

## Materials and methods

### Existing road detection methods

Road detection, a core task in intelligent transportation research ([1] [2]), has been studied for decades. Various models and methods based on different sensors are conducted to deal with this task in all kinds of scenes. [3] presents a survey of recent progress in road detection research. All models can be mainly divided into 2 trends:

1. approaches based on single sensor (monocular camera, stereo vision or lidar).
2. approaches based on combining multiple senors. ([4] [5])

It is reasonable to combine different inputs and construct a complete map of the environment. However, it is beyond the scope of this paper, in which we only talk about single input.

Among the single-camera based road detection methods ([6] [7] [8]), most of them are targeted to design classifiers, in which color ([9] [10]) and texture information ([11] [12]) are usually applied as main features. Lane marks ([13] [14]) are also frequently used on structured road detection, which are distinct especially on newly built asphalt road. However, these methods are sensitive to illumination or road shape. Meanwhile, flat plane assumption failure has significant impact on these models, especially in off-road scenes. The stereo-vision methods ([15] [16]) solves parts of these problems by employing the disparity map acquired by stereo matching. The U-V-disparity maps [17] and local descriptors [18] generated from disparity map can be used to analyze road regions [19]. Different from single-camera, stereo-vision can estimate distance through disparity maps. However, disparity error increases with distance, while dense stereo matching [20], needed by these models, are time consuming.

Compared to single-camera and stereo-vision, 3D lidar sensors outperform at measuring distance. Various methods are put forward on point cloud processing to deal with multifarious tasks, especially on object detecting and classifying ([21] [22]). Most of these models squash point cloud down to a 2D image-like format, because numerous processing methods based on image can be used after decades of researches on image processing. Recently, direct 3D data operating methods ([23] [24]) are studied by some scholars, but they are tentative and too time-consuming, which is not suitable to road detection task. Flat plane assumption ([25] [26]) is widely applied in 3D lidar based models. Flat areas are usually considered as road regions in high-definition lidar data. Road boundaries ([27] [28]) are also used frequently to locate road regions. Besides, a widely used road detection approach is proposed in [29] by estimating the min-max evaluation map. [30] presents a survey of grid map based road and obstacle detection methods for unmanned ground vehicles. Besides to grid map, projecting lidar points onto image plane ([31] [32]) is another expression of lidar data. In approach [33], lidar-histogram is proposed, which helps detect road in lidar-imagery. These well-known methods are mainly based on height difference between road and non-road regions. If the height difference is not salient, such as in off-road scenes, clearer data organization and stabler features are expected.

### Approach

To overcome the feedbacks of existing lidar based methods, as well as satisfying real-time requirements, we rearrange point cloud data, based on which a road consistency map and an obstacle map are generated. Road consistency map takes advantage of spatial relationship among point cloud, while obstacle map makes full use of height difference between road and non-road regions. Combining these two confidence maps, road region could be well segmented both in structured urban and unstructured rural scenes.

#### A. Rearrangement of lidar data

In order to deal with road detection task, we rearrange lidar data at first step to accomplish a presentation mode with spatial relationship and low complexity. Here, we use Velodyne HDL-64 S2 lidar, which is a core 3D lidar sensor of autonomous driving vehicles. It contains 64 laser heads, which are separated into 2 blocks with 32 lasers respectively. All laser heads are settled on a spinning base, providing a 360° horizontal field of view and 26.8° vertical field of view. The original raw output data of lidar are arranged on spherical coordinate system, mainly including rotation angle *θ*, measurement distance *d*, and radiation intensity *I*. Calibration file provided by manufacturer contains the pitch angle *ϕ* of each laser head. When we convert the (*d*, *θ*, *ϕ*) in spherical coordinate system to the (*x*, *y*, *z*) in Cartesian coordinates, approximately 1.33 million sampling points are detected per second, so it is quite necessary to rearrange the 3D lidar data before processing.

We construct an array of structures to store lidar data, in which each structure contains all information of one sampling point, including its coordinates (*x*, *y*, *z*), intensity *I*, which scan line it belongs to (*ScanID*) and the point ranking of such point (*PointID*) in this scan line. Here, we convert a point in spherical coordinate to Cartesian coordinates by

$$\begin{array}{c} \left\{ \begin{array}{cc} x & =d\text{cos}\phi \text{cos}\theta \\ y & =d\text{cos}\phi \text{sin}\theta \\ z & =d\text{sin}\phi \end{array}\right. \end{array}$$ (1)

**Fig 1:**
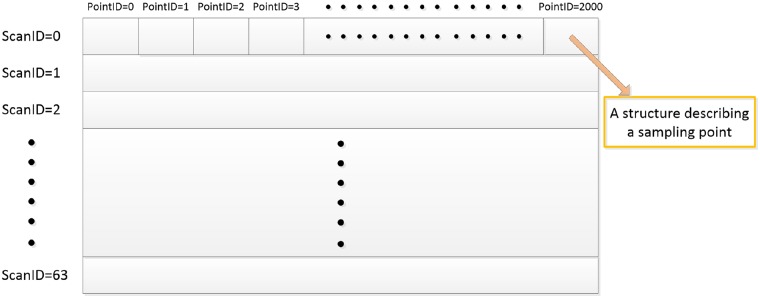
Array of structures.

*ScanID* indicates which laser head does the sampling point belong to. It ranks from 0 to 63 based on the pitch angle *ϕ* of the corresponding laser head. When lidar is settled on a big flat plane, No.0 scan line indicates the nearest one from lidar, while No.63 is the farthest one. *PointID* is concerned to the rotation angle *θ* of this point. Here, we define one lap of lidar data as a frame, and the rotating speed of lidar is settled at 10*Hz*. Then, a frame of lidar data contains 64 scan lines with approximately 2000 sampling points in each line, because the angular resolution of Velodyne HDL-64 S2 is 0.18° at 10*Hz*. Moreover, we use *ScanID* and *PointID* to arrange our array of structure. Our lidar data array is constructed as Fig 1. Each structure in this array represents a point, including 7 different features, *x*, *y*, *z*, *d*, *I*, *ScanID* and *PointID*, where *x*, *y*, *z* indicate the point location in Cartesian coordinate system, while *d* represents the linear distance, and the last 2 features describe adjacent relationship among points. Here, in our method, we mainly use the spatial information from lidar, so intensity *I* is kept but not used.

#### B. Consistency map

Road can be divided into 2 types simply, structured and unstructured roads. Normally, the structured roads have clear borders or strong difference with non-road regions, while the unstructured ones share weak natural edges with non-road regions. So, it is not quite reasonable to divide road regions simply based on boundary in all instances. In that case, we put forward a new pattern, distance-between-lines, to describe road region, which takes road undulating into account by using neighbor relationship among point cloud. No matter structured roads or unstructured ones, neighbor relationship is stable in both cases. However, unlike image presentation, it is quite difficult to discover neighbor relationship between scan lines by original 3D lidar data. Here, we witness an advantage of our rearrangement of lidar data, because sampling points in the same column have similar rotation angle, constructing spatial relationship between lines.

**Fig 2:**
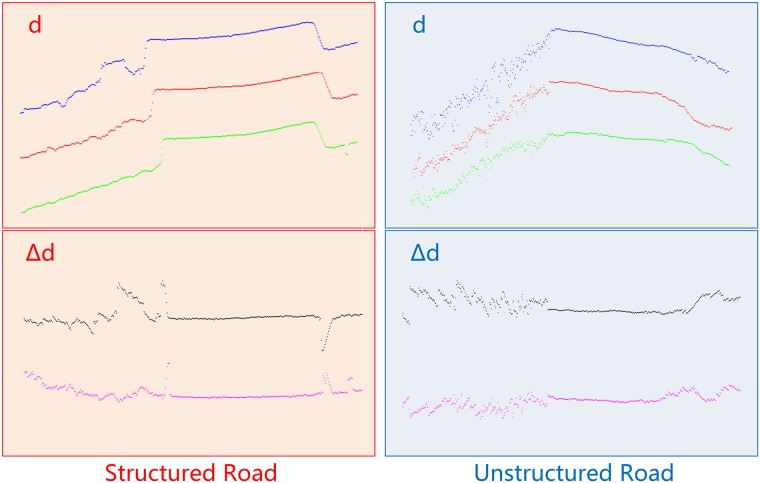
*d* and Δ*d* on structured and unstructured roads.

During our unstructured road experiments, we found that although road region become undulating in country road scenes, it shows a similar trend between scan lines along the road, which also occurs on structured roads (Fig 2). In Fig 2, three adjacent scans from structured road and three from unstructured are shown in two columns. It is clear to observe that 3 adjacent scans are similar, no matter in structured roads or unstructured ones. We call this consistency of a road. Then, we compute the distance between scans to smooth the road region, while amplify the chaos in non-road regions. The black scan in Fig 2 indicates the Δ*d* computed by the blue one minus the red one, while the purple scan comes from subtracting the green scan from the red one. Here, we witness that a much clearer road region could be segmented from each scan both in structured and unstructured road scenes. So, based on our structure array, we put forward a distance-between-lines feature to describe road consistency. Distance-between-lines (Δ*d*) is defined as follows.

$$\Delta d_{i}=\left|d_{ij}-d_{ik}\right|$$ (2)

Here, parameter *i* indicates *PointID*, while *j* and *k* represent *ScanID*, which are next to each other. *d* represents the distance from lidar center to such point. Using Δ*d*_*i*_, we reduce the impact of ups and downs on unstructured road, while remain or even enhance the messy situation on non-road regions. We suppose that road is always flatter than non-road regions in a scene, which means the variance of Δ*d* on road region is relatively smaller than those on non-road regions. This is because *d* values are in chaos on grass, rocks and so on, which lead to Δ*d*_*i*_ also in a jumble on these areas. As the *PointID* is arranged by rotation angle *θ*, structured edges will be amplified by Δ*d* rater than suppressed, unless lidar is right over the edge. We plot 3 neighbor scan lines from structured and unstructured roads on Fig 2, in which we can clearly see that, after computing Δ*d* between scans, the road region is much clearer to extract in both types of road. Moreover, the non road part witness more chaos, due to superposition of disorder.

In order to find candidate road regions, we separate each scan line into parts with suitable length by finding the inflection points. We use a low computational complexity way to locate the inflection points on each scan by the formula below:

$$|\Delta d_{i}-\sum_{j=i-k}^{i+k}W_{j}\times \Delta d_{j}|>thre$$ (3)

where

$$W_{j}=\frac{\text{exp}(-\frac{{\left(j-i\right)}^{2}}{2\sigma^{2}})}{\sum_{j=i-k}^{i+k}\text{exp}\left(-\frac{{\left(j-i\right)}^{2}}{2\sigma^{2}}\right)}$$ (4)

*i* and *j* indicate *PointID*, and *k* is filter radius. Here, we use the Gaussian filtering consequence as an adaptive interface, and inflection points are defined as those whose distance from the interface exceed threshold. To simplify operation, Gaussian template is used. Next, we use minimum-variance to evaluate the suitable point sequences on each scan line. Loss function is defined as:

$$L=\frac{1}{n-m}\sum_{i=m}^{n}∥\Delta d_{i}-\bar{\Delta d}∥$$ (5)

*m*, *n* indicate the inflection points located before, which determine a point sequence on a scan line. $\bar{\Delta d}$ is the average value of Δ*d*_*i*_. Here, in order to find all suitable *m*, *n* on each line, we could limit the road width by transcendental knowledge. Moreover, the value of L is also limited by a empirical threshold to select candidate road parts from each scan line.

**Fig 3:**
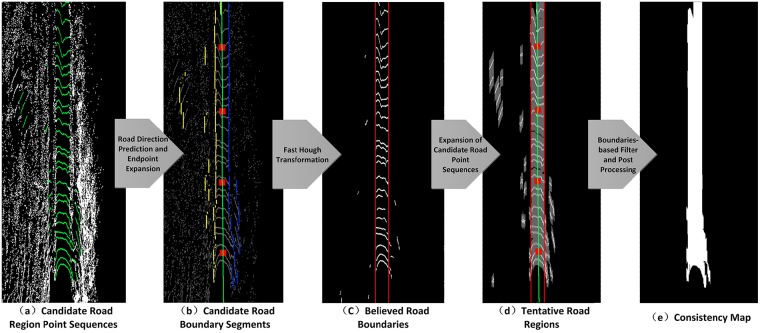
Consistency map $M_{Consistency}$ generating flow.

After locating the candidate point sequences on each scan, we project point cloud to a grid map shown in Fig 3a (candidate point sequences are colored in green), and the endpoints of such sequence are supposed to be candidate road boundary points in the grid map. Due to sparsity of lidar scans, we expand the endpoints on each scan along forward direction, then the end points of road regions become vertical line segments (Fig 3b). However, when we encounter to a line segment, it is not clear that it belongs to a left boundary or a right one, because lidar scan lines are circles in the grid map. Therefore, we predict the road direction by finding the midpoint of the longest continuous suitable point sequence on each scan in front of lidar. Moreover, in order to reduce computation, we choose 4 scans, instead of computing all 64 scans to predict the road direction. Just as the green line in Fig 3b, it is the road direction we predict, and the red squares are the 4 midpoints we choose. By road direction, we can easily decide the left boundary points (shown in yellow in Fig 3b) and the right ones (shown in blue in Fig 3b). Then, fast Hough transformation is adopted to determine the best lines, which go through most left or right boundary points, as the left and right boundary respectively (Fig 3c). Next, we expand all candidate road point sequences on each scan along forward direction as shown in gray in Fig 3d. The two road boundaries and the road direction could help to locate the true road regions from the candidate ones. Here, we believe that all gray pixels between boundaries are true road area, and the gray pixels linked to true road area but outside the detected boundaries are also believed to be road region. To polish up the outcome, we fill in the small blanks among gray pixels. Due to the sparsity of point cloud, we expend the farthest road region (with largest *ScanID*) along the nearest boundary direction to borders of the grid map, where is no laser echo. Finally, we acquire an image of road region by consistency measurement, which is called consistency map $M_{Consistency}$ here (Fig 3e). In case of transformation from BEV to perspective, we store the corresponding height of each pixel in $M_{Consistency}$, coming from our array of structures.

#### C. Obstacle map

Based on the novel array of structures mentioned in subsection A, a grid map is constructed by projecting 3D points to BEV, uniquely using *ScanID* density as the value of each grid. This coordinate system is image-like and gray-level of a pixel (*x*, *y*) represents the number of different *ScanID* that this grid contains. Dimensionality reduction from 3D to 2D and resolution reduction by generating grids hugely decrease computation afterwards, which is extremely significant to autonomous driving.

**Fig 4:**
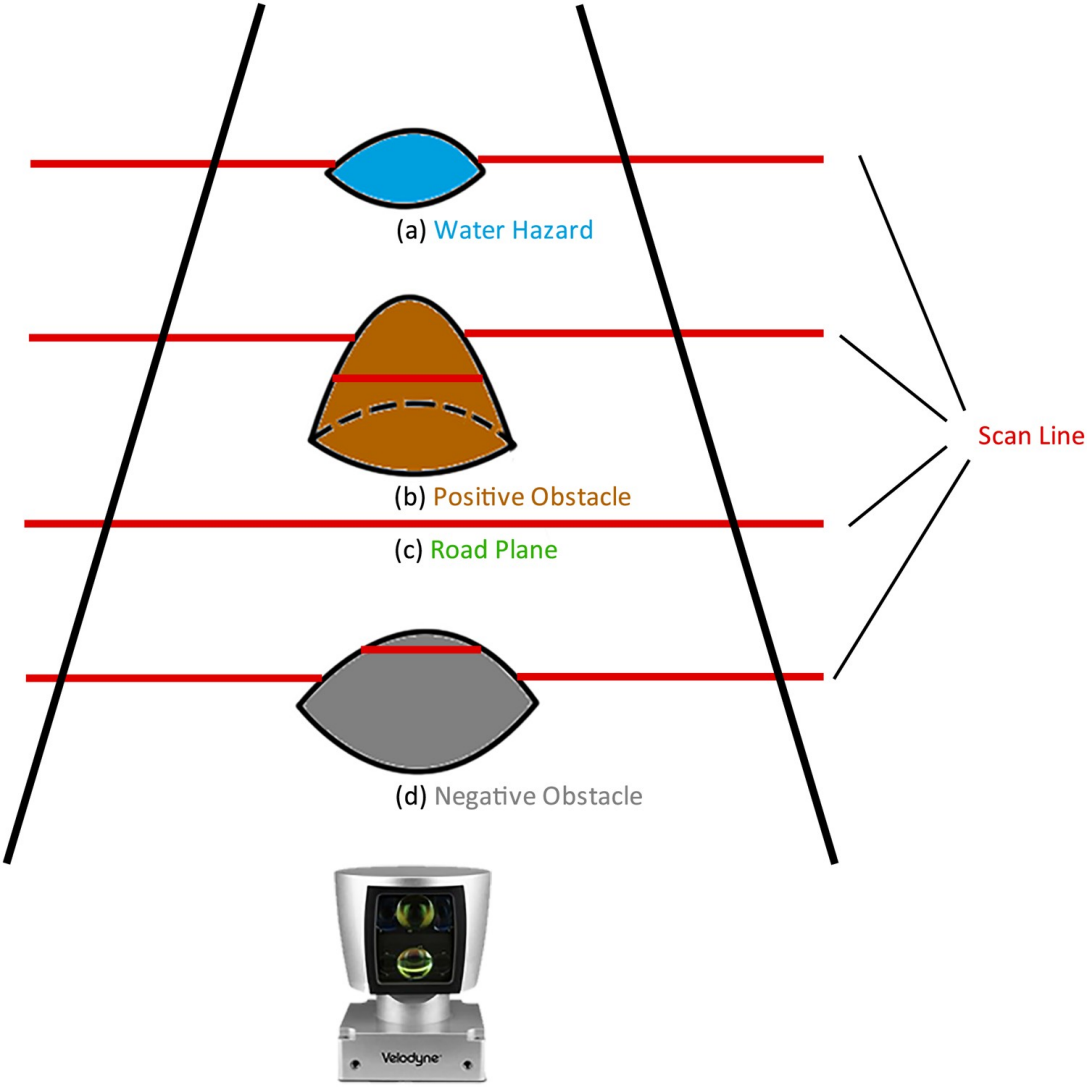
Different types of obstacles detected on road.

Here, instead of measuring the height of each grid, which is sensible to shaking and parameter calibration, we use another pattern to describe obstacles. According to our experiments, we discover a phenomenon that different types of obstacles on road causes different effects on scan lines, as can be seen in Fig 4. When scanning a road plane, the measured distances on one scan share nearly the same value. However, when points encounter a positive obstacle, the measured distances of these points are shorter than those on road plane. Oppositely, the distances from points on negative obstacles to laser head are longer. Water hazards will cause specular reflection, refraction and absorption on laser pulse, which leads to an undetected region without signal received by the lidar receiver. Dangerous water hazards on road always occur in a form of negative obstacle, so we mainly target on detecting positive and negative obstacles in this paper.

**Fig 5:**
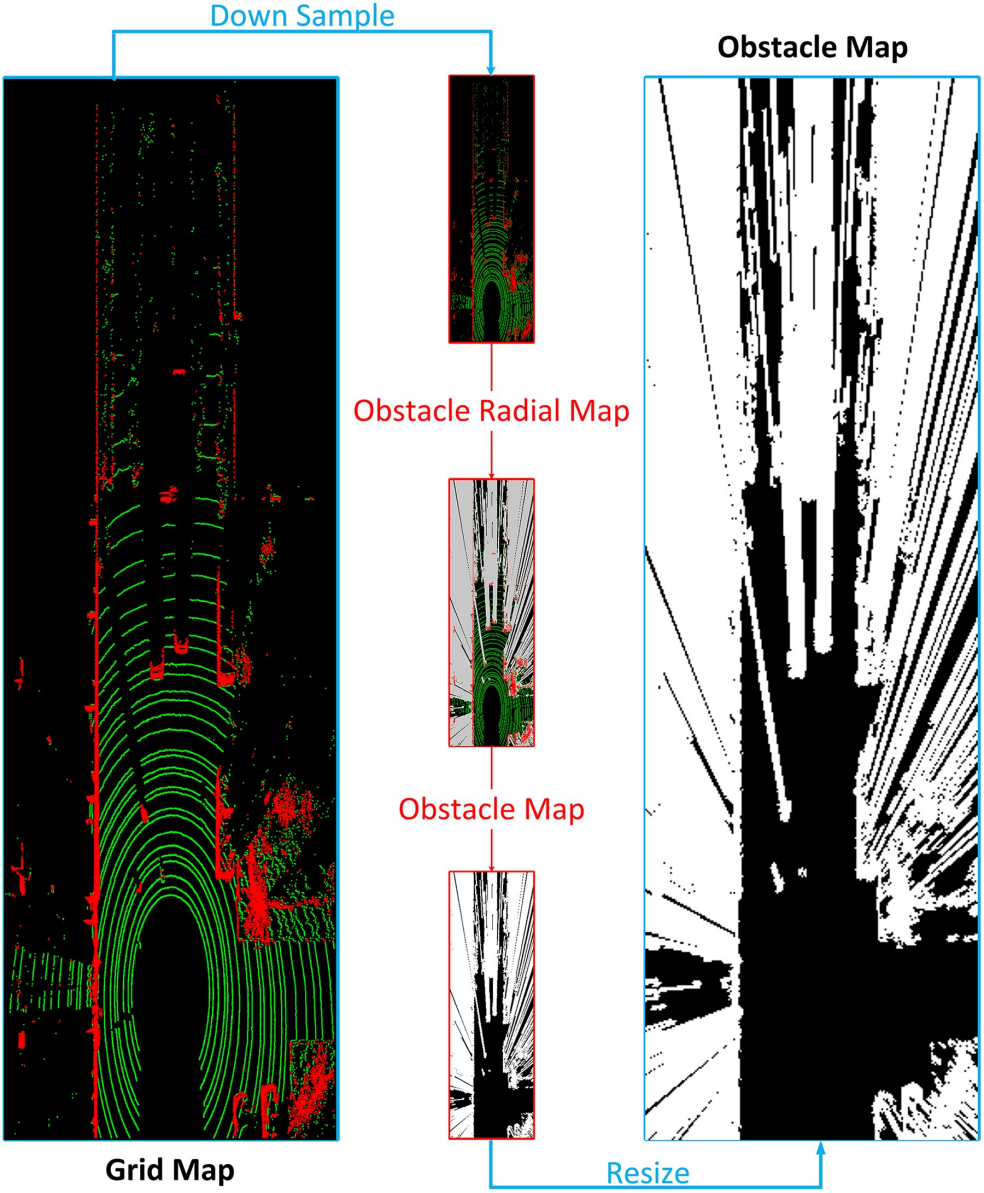
Obstacle map generating procedure.

According to Fig 4, lidar scan lines witness distortions when crossing positive and negative obstacles. The distance between two scan lines becomes much more narrow at obstacles. Furthermore, several lines could even gather at the back edge of negative obstacles and the front of positive ones. Here, we put forward a new pattern called *ScanID* density, which indicates the number of different *ScanID* in one grid. We use this pattern to generate an obstacle grid map to describe positive and negative obstacles at the same time. As the grid map in Fig 5 shown, pixels in green represent laser points in this grid are from the same scan line, while red pixels indicate that there are laser points from different scan lines in one grid. So, obstacles are easily located on pixel level in the grid map, in which red pixels are obstacles and green ones indicate relatively flat plane, while black regions are undetected areas.

As we all known, rays propagate along a straight line, so there are always undetected regions (shown in black in Fig 5) behind the obstacles (shown in red). The true road region in a perspective is the final target of our road detection model, and pixels of an obstacle in camera view including the red pixels and the black region behind in the corresponding grid map. So we introduce an obstacle radial map to describe obstacles in the grid map, which is also in conformity with BEV form. The obstacle radial map is generated by Algorithm 1.

**Algorithm 1** generating obstacle radial map $M_{Radial}$

**Input**: grid map $M_{Grid}$.

**Output**: obstacle radial map $M_{Radial}$.

1: **for** each *θ* ∈ (−90°, 90°) **do**

2:  *Flag* ← *false*;

3:  **for** each *x* from lidar center (*x*_0_, *y*_0_) to $M_{Grid}$ boundaries **do**

4:   *y* = tan *θ* × (*x* − *x*_0_) + *y*_0_;

5:   **if** $M_{Grid}\left(x,y\right)=red$ **then**

6:    *Flag* ← *true*; *Continue*;

7:   **else if** $M_{Grid}\left(x,y\right)=green$ **then**

8:    *Flag* ← *false*; *Continue*;

9:   **end if**

10:   **if** *Flag* **then**

11:    $M_{Grid}\left(x,y\right)=gray$;

12:   **end if**

13:  **end for**

14: **end for**

To sum up, we search all directions along radial rays from lidar center, and classify obstacle pixels and undetected pixels behind to obstacle regions. In order to accelerate obstacle radial map generating and filter the input simultaneously, we down sample the input grid map by 16 times. Next, obstacle regions, the red and gray pixels, are extracted from the obstacle radial map. Then, after a resizing procedure, the obstacle map $M_{Obstacle}$ is finally obtained. The obstacle map generating procedure is shown in Fig 5.

#### D. Combination and refinement

**Algorithm 2** refinement for the fused map $M_{Fused}$

**Input**: $M_{Fused}$.

**Output**: refined fused map $M_{Fused}$.

 1. $M_{Fused}=\text{PROJECTION}\left(M_{\text{Fused}}\right)$ → project to perspective

 2. $M_{Fused}=\text{ROI}\left(M_{\text{Fused}}\right)$

 3. $M_{Fused}=\text{ADJUST}\left(M_{\text{Fused}}\right)$ → spatial morphological filtering

 4. Keep the regions which reach the bottom, left or right border of the map $M_{Fused}$

 5. $M_{Fused}=\text{HOLE}-\text{FILL}\left(M_{\text{Fused}}\right)$

In order to obtain the ultimate road region pixels, based on which we can decide accessible area in autonomous driving tasks, two maps determined by distance-between-lines feature and obstacle detection respectively are fused to one, which makes full use of both road plane information and obstacle information simultaneously. The fused binary map is named as $M_{Fused}$, which is defined as follows.

$$M_{Fused}=M_{Consistency}\cap {\bar{M}}_{Obstacle}$$ (6)

**Fig 6:**
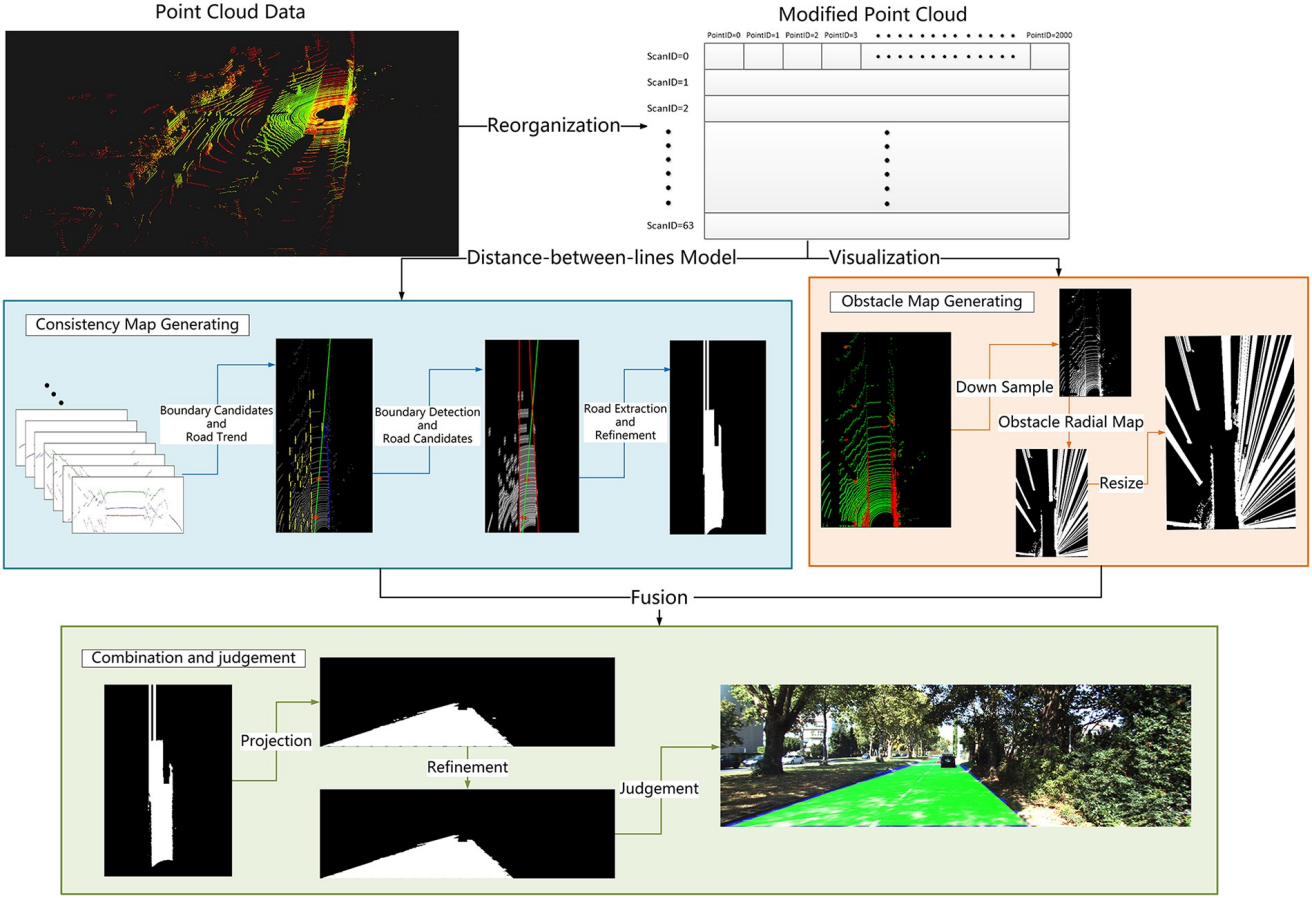
Processing flow.

Next, we project $M_{Fused}$ to perspective for further refinement and evaluation. The details of refinement procedure is shown in Algorithm 2. Besides, the whole processing flow is shown in Fig 6.

## Results

In this section, we evaluate the proposed method with 5 well-performed models demonstrated on KITTI dataset [34], including Road Estimation with Sparse 3D Points From Velodyne (RES3D-Velo) [31], Graph Based Road Estimation using Sparse 3D Points from Velodyne (GRES3D+VELO) [35], CRF based Road Detection with Multi-Sensor Fusion (FusedCRF) [36], LidarHistogram (LidarHisto) [33], Hybrid Conditional Random Field (HybridCRF) [37]. To easily express our method, we name it RDR, which indicates a Road Detection Method based on Reorganized Lidar Data.

### A. Dataset

In order to evaluate our proposed method, experiments are conducted on a PC by C/C++ with a CPU (2.5GHz) and a 8GB size memory without any code optimization, operated on a public dataset (KITTI). KITTI road dataset contains 289 marked and 290 unmarked road scenes for method evaluation.

We use the same evaluation criteria as KITTI, including MaxF (Maximum F1-measure), AP (Average Precision), PRE (Precision), REC (Recall), FPR (False Positive Rate), FNR (False Negative Rate), to compare our method with other models. The definition of MaxF, PRE, REC, FPR and FNR are as follows:

$$PRE=\frac{\left|M\cap G\right|}{\left|M\right|},REC=\frac{\left|M\cap G\right|}{\left|G\right|},$$ (7)

$$MaxF=\text{max}(\frac{2\times PRE\times REC}{PRE+REC})$$ (8)

$$FPR=\frac{number\;of\;false\;positives}{number\;of\;negative\;instances},FNR=\frac{number\;of\;false\;negatives}{number\;of\;positive\;instances}$$ (9)

where M indicates a binary segmentation, while G represents the ground truth of such instance.

### B.Comparison

**Fig 7:**
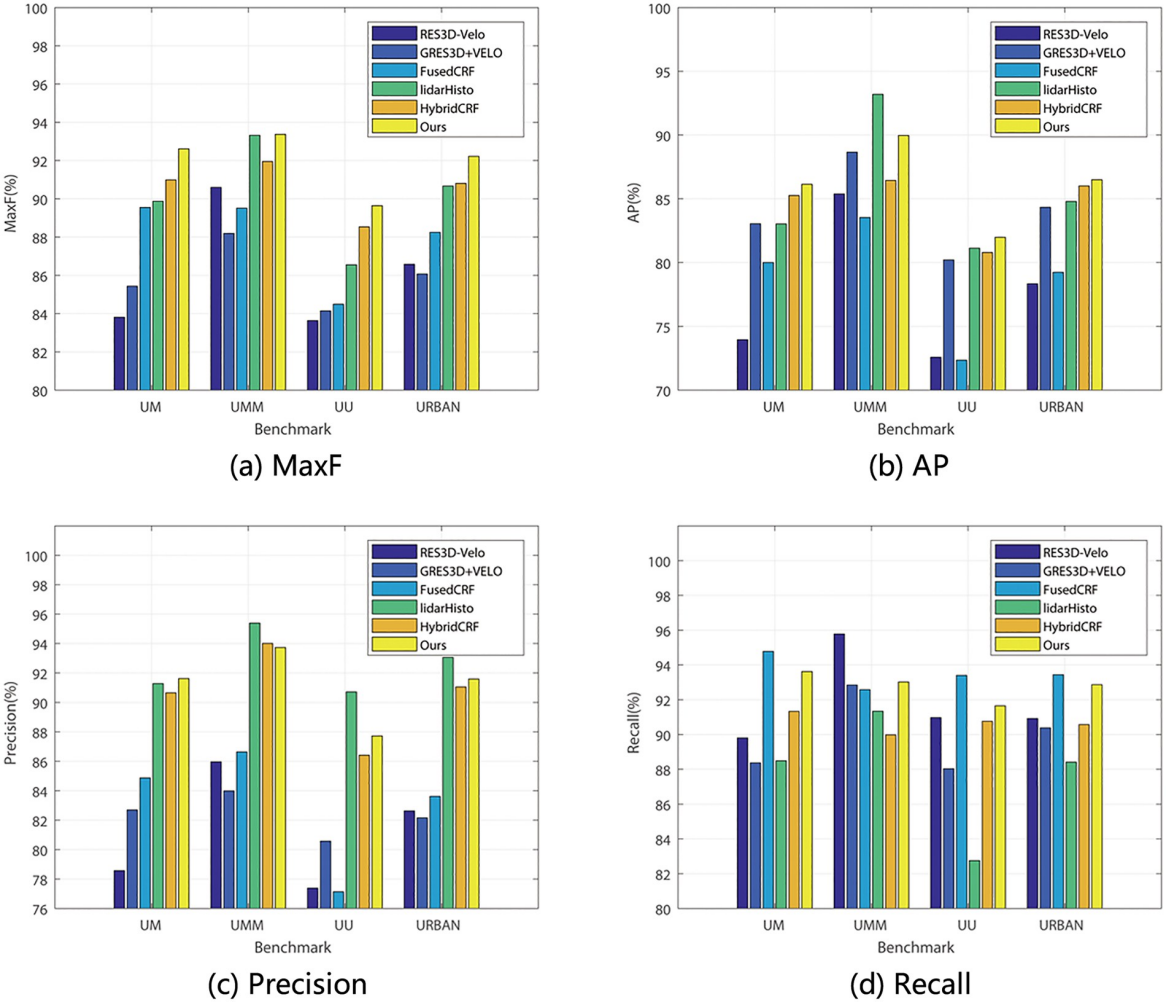
Bar charts of MaxF, AP, Precision and Recall comparison.

In order to prove the efficiency of our method, we compare it with other road detection models on KITTI dataset, which utilize lidar information, including RES3D-Velo [31], GRES3D+VELO [35], FusedCRF [36], LidarHisto [33], HybridCRF [37], to evaluate the validity of our method. A performance comparison is shown in Table 1 and bar charts of significant indexes (Fig 7) are also proposed for clearer observation. According to Table 1, our method generates much more accurate outcomes than other models on UM_Road, UU_ROAD and URBAN_ROAD benchmarks. On UMM_ROAD benchmark, LidarHisto performs well and almost catch up with RDR, lagging behind 0.05% only.

**Table 1:** Performance comparison with other methods on KITTI.

Method	Benchmark	MaxF	AP	PRE	REC	FPR	FNR
RES3D-Velo	UM_ROAD	83.81%	73.95%	78.56%	89.80%	11.16%	10.20%
	UMM_ROAD	90.60%	85.38%	85.96%	95.78%	17.20%	4.22%
	UU_ROAD	83.63%	72.58%	77.38%	90.97%	8.67%	9.03%
	URBAN_ROAD	86.58%	78.34%	82.63%	90.92%	10.53%	9.08%
GRES3D+VELO	UM_ROAD	85.43%	83.04%	82.69%	88.37%	8.43%	11.63%
	UMM_ROAD	88.19%	88.65%	83.98%	92.85%	19.48%	7.15%
	UU_ROAD	84.14%	80.20%	80.57%	88.03%	6.92%	11.97%
	URBAN_ROAD	86.07%	84.34%	82.16%	90.38%	10.81%	9.62%
FusedCRF	UM_ROAD	89.55%	80.00%	84.87%	94.78%	7.70%	5.22%
	UMM_ROAD	89.51%	83.53%	86.64%	92.58%	15.69%	7.42%
	UU_ROAD	84.49%	72.35%	77.13%	93.40%	9.02%	6.60%
	URBAN_ROAD	88.25%	79.24%	83.62%	93.44%	10.08%	6.56%
lidarHisto	UM_ROAD	89.87%	83.03%	91.28%	88.49%	3.85%	11.51%
	UMM_ROAD	93.32%	93.19%	95.39%	91.34%	4.85%	8.66%
	UU_ROAD	86.55%	81.13%	90.71%	82.75%	2.76%	17.25%
	URBAN_ROAD	90.67%	84.79%	93.06%	88.41%	3.63%	11.59%
HybridCRF	UM_ROAD	90.99%	85.26%	90.65%	91.33%	4.29%	8.67%
	UMM_ROAD	91.95%	86.44%	94.01%	89.98%	6.30%	10.02%
	UU_ROAD	88.53%	80.79%	86.41%	90.76%	4.65%	9.24%
	URBAN_ROAD	90.81%	86.01%	91.05%	90.57%	4.90%	9.43%
RDR (ours)	UM_ROAD	92.61%	86.14%	91.62%	93.62%	3.90%	6.38%
	UMM_ROAD	93.37%	89.97%	93.73%	93.02%	6.84%	6.98%
	UU_ROAD	89.64%	81.98%	87.72%	91.65%	4.18%	8.35%
	URBAN_ROAD	92.22%	86.50%	91.59%	92.87%	4.70%	7.13%

Our method has a novel outcome compared with models demonstrated on KITTI dataset.

**Fig 8:**
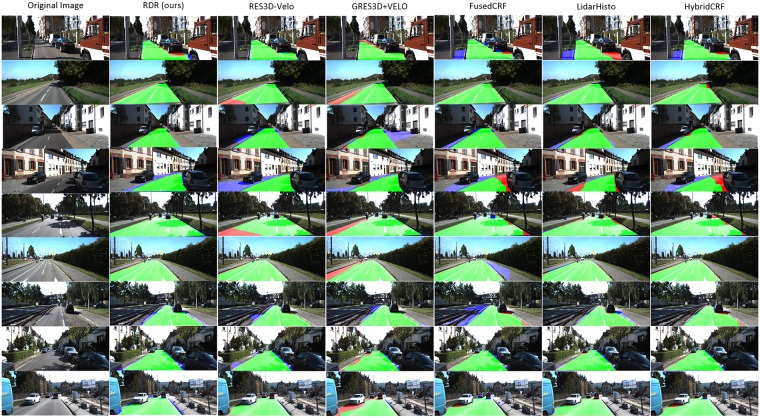
Visual results comparison.

**Fig 9:**
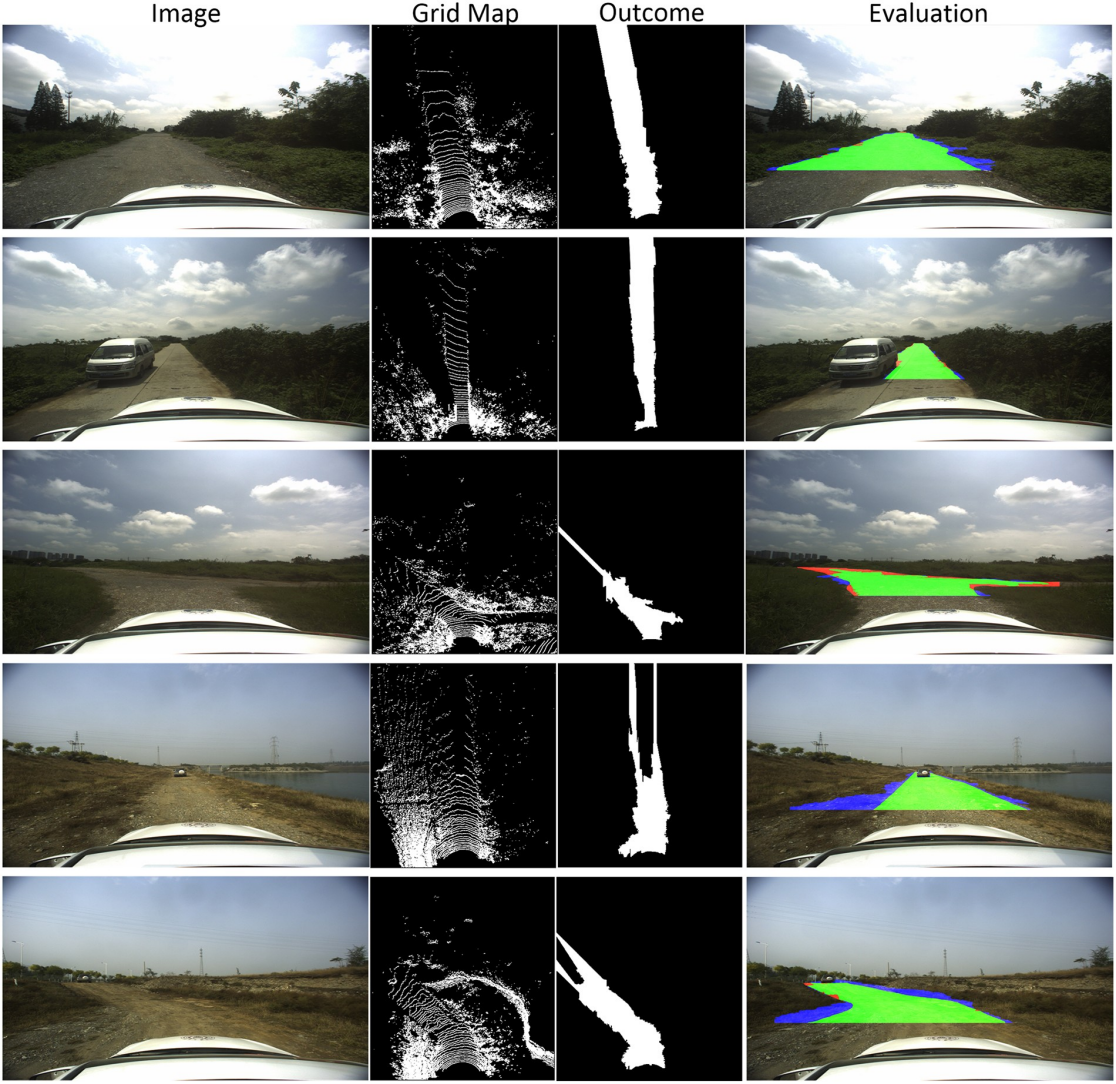
Visual results of RDR on unstructured road.

The time efficiency comparison has been shown in Table 2, in which our method performs the first place among the methods listed. In general, our model obtains a state-of-art result on KITTI dataset with low time cost. The typical visual results of these models are also listed in Fig 8. To further evaluate the efficiency of RDR on unstructured road, experiments are taken on the data acquired by ourselves. Real vehicle tests are carried out, and 30 typical rural road scenes are chosen to form a small dataset with road region tags. Our model achieves 84.47%, 81.79%, and 87.33% on MaxF, PRE and REC respectively on this dataset. The typical visual results of rural road scenes are proposed in Fig 9.

**Table 2:** Time efficiency comparison.

Method	Runtime	Environment
RES3D-Velo	360ms	1 core @ 2.5 Ghz (C/C++)
GRES3D+VELO	60ms	4 cores @ 2.8 Ghz (C/C++)
FusedCRF	2000ms	1 core @ 2.5 Ghz (C/C++)
lidarHisto	100ms	2 core @ 2.5 Ghz (C/C++)
HybridCRF	1500ms	1 core @ 2.5 Ghz (C/C++)
RDR (ours)	60ms	2 core @ 2.5 Ghz (C/C++)

Our method outperforms other models demonstrated on KITTI dataset on time efficiency.

## Discussion

**Fig 10:**
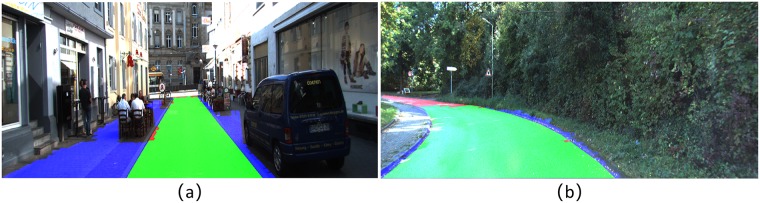
Fail instances.

Although RDR performs well and exceeds the compared models on datasets, it does fail in some cases. These failures are mainly caused by two attributes of our method. Fig 10 shows the typical failure cases collected from KITTI dataset. Fig 10(a) is mainly caused by failure of assumption 1. The non-road region is a flat plane, and there is no inflection point at road boundaries, so that RDR select a point sequence containing both road and non-road regions as one candidate road part. Fig 10(b) occurs because we use straight line fitting to locate road boundaries, which are used for choosing road region from candidates in RDR. To reduce this influence, we increase the vertical resolution in grid map to stretch road along forward direction and reduce bend radian. Fig 10(b) is the outcome after adopting this step.

## Conclusion

Throughout this paper, we present a novel road detection method based on modified lidar data, which meets real-time requirement. The reorganization of lidar data constructs spatial relationship based on *ScanID* and *PointID*, which are actually quantifications of laser head pitch angle and lidar rotation angle to some extent. Based on the reorganized lidar data, two main innovations of our work, distance-between-lines and *ScanID* density, are put forward to generate consistency map and obstacle map respectively. Next, two maps are fused to one, and after refinement, road region is detected ultimately. Experimental results indicate a novel performance of our proposed model.

With regard to future work, multi-sensor fusion is under consideration, which can make environment modeling much more perfect. For example, failure in Fig 10(a) could be solved, if camera input is combined. Furthermore, a certain degree of information redundancy ensures the safety of autonomous vehicle. Second, we intend to use lidar information more deeply. Dimensionality reduction and ignorance of intensity abandon a lot of precious information from point cloud. So that, employing intensity and making use of point cloud directly would be our focus of attention afterwards.
